# Chest CT-scan finding of asymptomatic COVID-19 pneumonia: a prospective 542 patients’ single center study

**DOI:** 10.11604/pamj.2020.36.257.23632

**Published:** 2020-08-07

**Authors:** Asma Achour, Oussema Dkhil, Jamel Saad, Mabrouk Abdelali, Ahmed Zrig, Badii Hmida, Mondher Golli, Mezri Maatouk, Walid Mnari

**Affiliations:** 1 Department of radiology A, Fattouma Bourguiba University Hospital Monastir, Tunisia and Monastir University of Medicine, Monastir, Tunisia

**Keywords:** SARS-CoV-2, COVID-19, asymptomatic, computed tomography, ground glass opacity

## Abstract

Since asymptomatic infections as “covert transmitter”, and some patients can progress rapidly in the short term, it is essential to pay attention to the diagnosis and surveillance of asymptomatic patients with SARS-COV2 infection. CT scan has great value in screening and detecting patients with COVID-19 pneumonia, especially in the highly suspected or probable asymptomatic cases with negative RT-PCR for SARS-COV2. This study aimed to detect incidentally COVID-19 pneumonia on medical imaging for patients consulting for other reasons.

## Introduction

In the worldwide COVID-19 outbreak, asymptomatic infected individuals may become the contagious source of SARS-CoV-2; some of them progress rapidly to acute respiratory distress syndrome and systemic complications. Early detection has become crucial to ensure rapid prevention and timely treatment. So numerous healthcare professionals are involved in responding to the health crisis mostly with unknown infected patients. Chest CT imaging of asymptomatic cases with COVID-19 pneumonia has definite characteristics and the role of radiologists has been emphasized [1,2]. The definitive diagnosis of COVID-19 relies on real-time Reverse Transcription-Polymerase Chain Reaction (RT-PCR) on a nasopharyngeal swab or other respiratory specimens. However, the sensitivity of RT-PCR depends on several factors, including the quality of the sampling, and the viral burden at the time of specimen collection, explaining that some cases may be missed [2,3]. In the current emergency, the low sensitivity of RT-PCR implies that many COVID-19 patients may not be identified and may not receive appropriate treatment in time; such patients constitute a risk for infecting a larger population given the highly contagious nature of the virus. Chest CT, as a routine imaging tool for several pathologies, is relatively easy to perform and can produce fast diagnosis of COVID-19 pneumonias with characteristic findings including ground-glass, opacities, multifocal patchy consolidation, and/or interstitial changes with a peripheral distribution [3]. Given the current prevalence of the disease, we can expect incidental detection of COVID-19 pneumonia on examinations not directly performed for this reason. This situation is of critical importance since radiologically visible COVID-19 pneumonia is associated with potential virus transmission [4,5]. In this study we aimed to detect incidentally COVID-19 pneumonia on medical imaging for patients consulting for other reasons.

## Methods

From March 15 to May 10, 2020, 542 patients in Monastir, Tunisia who had a CT scan for different indications and who had a screening chest CT scan at the same time of the requested examination even for acute abdominal pain as well as patients who did requested chest CT scan were included. Three radiologists (Resident in medical imaging during training and two radiologists with 15 years of experience in interpreting chest CT imaging), reviewed all chest CT images and decided on positive or negative CT findings by consensus. The epidemiological history and clinical presentation were available for all readers. The radiologists classified the chest CT as positive or negative for COVID-19. For each patient, the chest CT scan was evaluated by these following characteristics: (1) ground-glass opacities, (2) parenchymal consolidation, (3) peripheral position of ground-glass opacities and consolidation, (4) number of lobes affected, (5) degree of involvement of each lung lobe in addition to overall extent of lung involvement measured by means of a “total severity score” as detailed below, (6) presence of nodules (alveolar nodule or “tree in bud” pattern nodules), (7) pleural effusion, (8) thoracic lymphadenopathy (defined as lymph node size of ≥10 mm in short-axis dimension), (9) small airways abnormalities (wall thickening, bronchiectasis, and endoluminal secretions), (10) central distribution of disease (“peribronchovascular” predominant disease) and (11) presence of underlying lung disease such as emphysema or fibrosis. Other abnormalities, including linear opacities, opacities with a rounded morphology, opacities with a “reverse halo” sign, opacities with a “crazy-paving” pattern, and opacities with intralesional cavitation, were noted. Ground-glass opacification was defined as hazy increased lung attenuation with preservation of bronchial and vascular margins, whereas consolidation was defined as opacification with obscuration of margins of vessels and airway walls. Each of the five lung lobes was assessed for degree of involvement and classified as none (0%), minimal (1-25%), mild (26-50%), moderate (51-75%), or severe (76-100%). Patients with chest CTs classified as a COVID-19 suspect had RT-PCR tests and were isolated. For patients with negative RT-PCR tests but positive CT results, clinical follow-up compared with serial chest CT scans and a second RT-PCR were carried out to further confirm the imaging diagnosis.

## Results

542 patients (mean age, 45years [4,86]; Sex ratio 65% [357/542] men) were available for CT chest screening. Only nine patients (1.6 %) had positive CT scans and in which RT-PCR was made. Of 9 patients with positive CT scans results, only 22 % (2/9) had positive RT-PCR and 77% had negative test even for second RT-PCR. Of the 9 patients, five patients (55%) had ground-glass opacities and consolidation, four patients (45%) had only ground-glass opacities (with no consolidation). Two patients (22%) had opacities in one lobe, 6 patients (66%) had four affected lobes, and 1 patient (12%) had disease affecting all five lobes (Table 1). *Case 1 and 2 show an example of COVID-19 pneumonia incidentally discovered on chest CT. Case 3 shows an example of suspected coronavirus pneumonia but RT-PCR of SARS-CoV-2 result and other medical investigation redress the diagnosis*.

**Table 1:** characteristics of the included 542 patients

Characteristic	Results
**Mean age**	45 years Range of 4 to 86
**Male**	357/542
**Positive CT scans**	1.6% (9/542)
**Positive CT scans with positive RT-PCR**	22% (2/9)
**Findings and manifestations of chest CT Consistent with viral pneumonia**	1.6% (9/542)
**Ground-glass opacity**	100% (9/9)
**Consolidation**	55% (5/9)
**Reticulation/thickened interlobular septa**	1.6% (9/542)
**Peripheral position of ground-glass opacities and consolidation**	100% (9/9)
**No CT findings of viral pneumonia**	98.4%(533/542)

**Figure 1:**
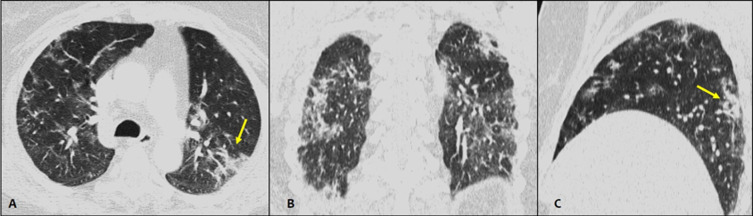
axial (A) coronal (B) and sagittal (C) CT images without intravenous contrast showing bilateral and subpleural pulmonary ground-glass predominant, associated with linear and sub segmental condensation (arrows)

**Case 1**: CT images without intravenous contrast performed in a 62-year-old Woman consulting in the emergency room for abdominal pain and vomiting. The abdominal scanner was without significant abnormalities. The CT scan showed bilateral and subpleural pulmonary ground-glass predominant. It is associated with linear and sub segmental condensation (Figure 1).A RT-PCR of SARS-CoV-2was performed and returned positive.

**Figure 2:**
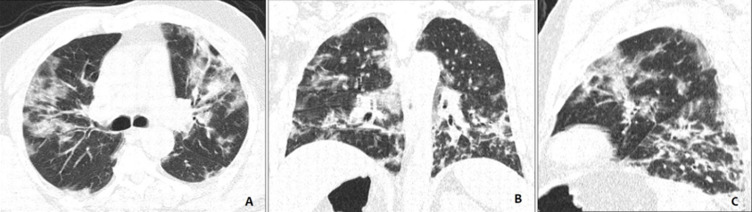
axial (A) coronal (B) and sagittal (C) CT images without intravenous contrast showing bilateral subpleural and peripheral ground-glass opacities with pulmonary condensation predominant in the lower lobes

**Case 2**: CT images without intravenous contrast performed in a 57-year-old men consulting in the emergency room for brain trauma following a loss of consciousness. The brain scanner was without abnormalities. The findings included bilateral subpleural and peripheral ground-glass opacities with pulmonary condensation predominant in the lower lobes (Figure 2). Two days later the patient presented a cough, chest pain and high fever (39°C). A RT-PCR of SARS-CoV-2 was performed and returned positive.

**Figure 3:**
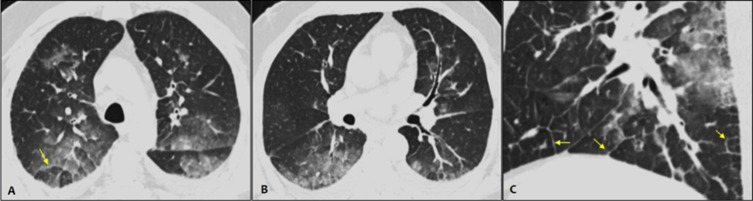
axial (A and B) and aggrandized right lower lobe sagittal (C) CT images without intravenous contrast showing bilateral and central ground-glass with broncho vascular bundle and interlobular septal thickening (arrows)

**Case 3**: CT images obtained without intravenous contrast in a 52-year-old male following a chest CT scan as part of exploration of atypical chest pain without dyspnea and normal levels of cardiac enzymes. The first ECG showed no repolarization abnormalities. The CT scan demonstrated bilateral and central ground-glass with Broncho vascular bundle and interlobular septal thickening (Figure 3). Interstitial pulmonary edema secondary to probably coronary syndrome was the most likely diagnosis. However, the patient was isolated and RT-PCR test was performed due to the epidemiologic context which proved negative. Meantime, the cardiac ultrasonography control ECG and evolution of cardiac enzyme level confirm the acute coronary syndrome with subacute pulmonary edema.

## Discussion

For the nine patients with chest CTs classified as a COVID-19 suspect, specific management was pursued, including the requirement to wear a mask, immediate information of the suspicious imaging findings to the patient and the referring physician, and activation of the RT-PCR test procedure, as well as careful disinfection of the CT suite. All these infection control measures aimed to prevent potential transmission to other individuals, including other patients who are potentially vulnerable, radiology staff, relatives, and any other personal contacts. During the COVID-19 outbreak, the radiology staff, especially radiology technicians, must be aware and trained to any incidental detection of appearances suggestive of SARSCoV-2 infection. Such findings may be discovered not only on chest CT but, during any imaging performed for other clinical situations, also when the lungs are partially visible in non-pneumological studies. Therefore, while specific tracks for suspected or known COVID-19 patients have been established, the healthcare team should keep in mind that asymptomatic or paucisymptomatic carriers are potentially present in the non-COVID-19 pathway. Several consequences flow from this. First, radiology technicians might be unexpectedly exposed and should be provided with adequate protective equipment. Second, we recommend reviewing all available chest images within any imaging examination as soon as possible before the patient leaves the radiology facility to identify suggestive abnormalities. Third, in case of suggestive findings, the CT suite needs to be appropriately cleaned before the next examination, while patients should be quickly directed to the COVID-19 pathway. Taken together, these actions are likely to slow down the clinical workflow, and prolonged waiting times for outpatients should be avoided [5]. Maintaining limited but adequate precautionary measures would be warranted to safeguard the health of the community, including healthcare actors and vulnerable persons. We suggest continuing to review chest images immediately after the acquisition to promptly detect suggestive features of COVID-19 pneumonia, even when the examination is performed for other clinical indications.

RT-PCR is currently considered as the gold standard diagnostic method for COVID-19. However, the sensitivity of this method in throat swabs in COVID-19 is around 59% [3]. To date, in several studies the sensitivity of chest CT has exceeded that of RT-PCR, and the authors emphasized the potential of chest CT as the primary screening tool for COVID-19 [3,6]. The sensitivity of chest CT is unquestionable and encouraged for cases where there is need to determine the extension of disease and alternative diagnoses. Chest CT is a conventional, non-invasive imaging modality with high accuracy and speed. Based on available data published in recent literature, almost all patients with COVID-19 had characteristic CT features in the disease process, such as different degrees of ground-glass opacities with/without crazy-paving sign, multifocal organizing pneumonia, and architectural distortion in a peripheral distribution [3,4]. In this study only two patients with typical CT manifestations had positive RT-PCR tests. In the context of emergency disease control, some false-positive cases may be acceptable. On the other hand, given the relatively low positive rate of RT-PCR assay, some “false-positive” cases on CT may indeed be “true-positive” if RT-PCR assay is an imperfect standard of reference. In fact, from the results of this study, 7 patients (1.3%) with negative RT-PCR results but positive chest CT scans were isolated and followed with a second RT-PCR performed to ensure the absence of sars-cov2 infection.

## Conclusion

This study showed a low incidence of patients with COVID-19 pattern pneumonia on daily CT scan activity outside the hospital specific pathway. However, this situation is of critical consequences since radiologically visible COVID-19 pneumonia is associated with potential severe complication for patients and potential virus transmission within the health staff. Of note, once the outbreak will be under control and the number of cases declines, these emergency measures will be progressively discontinued. At the same time, the spread of SARS-CoV-2 will probably not end overnight, and some cases will still be diagnosed in the following months.

### What is known about this topic

- The typical hallmarks of COVID-19 pneumonia reported on CT scans are characterized by bilateral lung involvement, multifocal ground-glass opacities, and consolidation in a typical peripheral with a posterior-dependent gradient and more consolidation in the postero-basal regions;
- Patients may have negative RT-PCR for COVID-19 at initial presentation despite chest CT findings typical of viral pneumonia.

### What this study adds

- Ground glass opacities, consolidation, reticular pattern, and crazy paving pattern are typical CT manifestations of COVID-19;
- CT manifestations may associate with the progression and prognosis of COVID-19;
- Chest CT is recommanded for screening for COVD-19 for patients with clinical and epidemiologic features compatible with COVID-19 infection particularly when RT-PCR testing is negative.
